# Recent advances in SmFe_12_-based permanent magnets

**DOI:** 10.1080/14686996.2021.1913038

**Published:** 2021-06-22

**Authors:** Y. K. Takahashi, H. Sepehri-Amin, T. Ohkubo

**Affiliations:** Research Center for Magnetic and Spintronic Materials, National Institute for Materials Science, Tsukuba, Japan

**Keywords:** Permanent magnet, ThMn_12_, Sm(Fe-Co)_12_, coercivity, 40 Optical, magnetic and electronic device materials, 203 Magnetics / Spintronics / Superconductors

## Abstract

To realize a sustainable society, ‘green technology’ with low (or even zero) CO_2_ emissions is required. A key material in such technology is a permanent magnet because it is utilized for electric-power conversion in several applications including electric vehicles (EVs), hybrid EVs (HEVs), and turbines for wind power generation. To realize highly efficient electric-power conversion, a stronger permanent magnet than Nd–Fe–B is necessary. One potential candidate is a Fe-rich SmFe_12_-based compound with a ThMn_12_ structure. In this paper, the phase stability, structure, and intrinsic and extrinsic magnetic properties in both film and bulk forms are reviewed. Based on these results, a possible way to realize a strong SmFe_12_-based permanent magnet in bulk form is discussed.

## Graphical abstract

**Figure:**
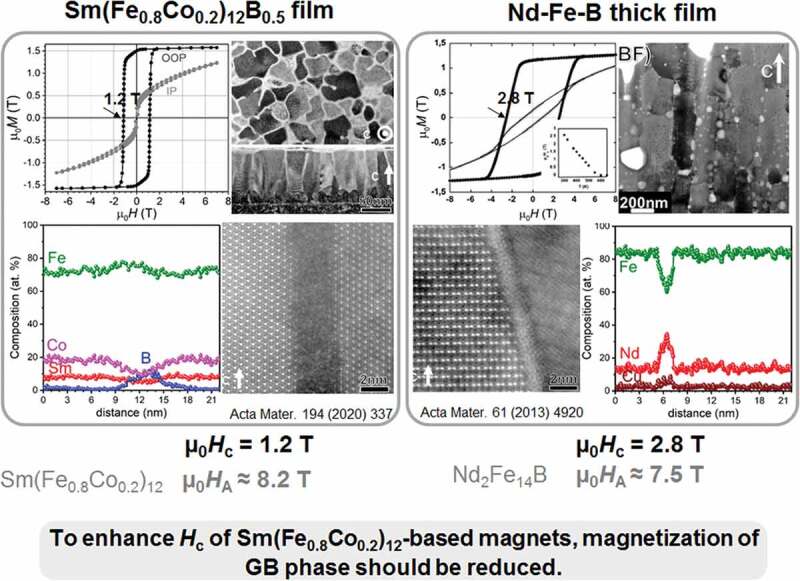

## 1. Introduction

With the rapid demand for ‘green technology’ to reduce CO_2_ emissions, the development of highly efficient electric motors and generators is required. For this, high-performance permanent magnets, especially for traction motors of EVs, HEVs, and turbines of wind power generation are necessary. Because the working temperature of the motor of EVs/HEVs is approximately 150°C, a high coercivity (μ_0_*H*_c_) larger than 0.8 T is necessary at this temperature. Although Nd–Fe–B is the most important permanent magnet for ‘green technology,’ one drawback is its poor thermal resistance to μ_0_*H*_c_ owing to its low Curie temperature (*T*_c_) [1]. Therefore, there is a strong demand for the development of new permanent magnets whose magnetic properties are superior to that of Nd–Fe–B, especially at elevated temperatures. One of the candidates for the next generation of permanent magnets is an RT_12_-based compound with a ThMn_12_ structure, where R and T represent rare-earth and transition elements, respectively.

RFe_12_-based magnets had received significant attention in the 1990s [2–8] because it was expected to show high saturation magnetization (μ_0_*M*_s_) owing to the higher molar fraction of Fe in the rare-earth compound, high magnetocrystalline anisotropy (*K*), and high *T*_c_. However, it has not received much attention as the next generation of permanent magnets because of several reasons. The first reason is the phase instability of the RFe_12_-based compound. To obtain the ThMn_12_ structure at room temperature, phase stabilizing elements such as Ti, V, Cr, Mn, Al, W, Si, Mo, and Zr [9–27] are necessary. These phase stabilizing elements reduce the magnetization. The second reason is the low μ_0_*H*_c_. To achieve a high μ_0_*H*_c_, reduction of the grain size and magnetic isolation of each grain and/or introducing pinning sites for magnetic domain wall motion is necessary. Therefore, a multiphase material with different *K* and/or μ_0_*M*_s_ is required for high μ_0_*H*_c_. Contrary to the Nd–Fe–B magnet, the RFe_12_-based compound did not show a high μ_0_*H*_c_. As shown in the phase diagram of the Sm–Fe–Ti system reported by Neiva et al. [28], there is no non-magnetic phase that can be equilibrated with the RFe_12_ phase. By analyzing the phase diagram, obtaining a sufficiently high μ_0_*H*_c_ in this system for permanent magnet application seems to be difficult. The advantage of Nd–Fe–B magnets is that Nd_2_Fe_14_B phase equilibrate with the Nd-rich phase, enabling liquid sintering process and formation of Nd-rich grain boundary phase which is needed to realize a high coercivity [29]. After the rare-earth element crisis in 2011, research interests in the development of rare-earth lean magnets or even rare-earth-free magnets have increased. Following this trend, there has been a revival in research on the RFe_12_ phase for permanent magnet applications.

To check the potential of the RFe_12_ phase as a permanent magnet, the intrinsic magnetic properties of the SmFe_12_-based epitaxial thin film without adding any phase-stabilizing element has been investigated [30,31]. By substituting Fe with Co, a large μ_0_*M*_s_, high anisotropy *K*, and high *T*_c_, which are superior to those of Nd_2_Fe_14_B, was obtained, and this showed the potential of these materials for room and elevated temperature applications [30]. In view of the material abundance, Sm is less abundant than Nd. At this moment, this may not be a big problem of considering Sm(Fe_0.8_Co_0.2_)_12_-based compounds for the next-generation medium/high performance permanent magnets, because Sm is a by-product in the purifying process of Nd. However, Co is another scarce element which is used in the Sm(Fe_0.8_Co_0.2_)_12_-based compound to have sufficiently large saturation magnetization. However, it is necessary to find a way to minimize the usage of Co in the future while maintaining a large saturation magnetization in the SmFe_12_-based compounds with ThMn_12_-type crystal structure.

In this paper, we first review the fundamentals of the RFe_12_ phase. Next, we show the potential of the SmFe_12_–based phase as the permanent magnet in a model experiment of a thin film and also discuss recent progress for bulk magnets.

## 2. Crystal structure and phase stability of RT_12_ phase

**Figure 1.**
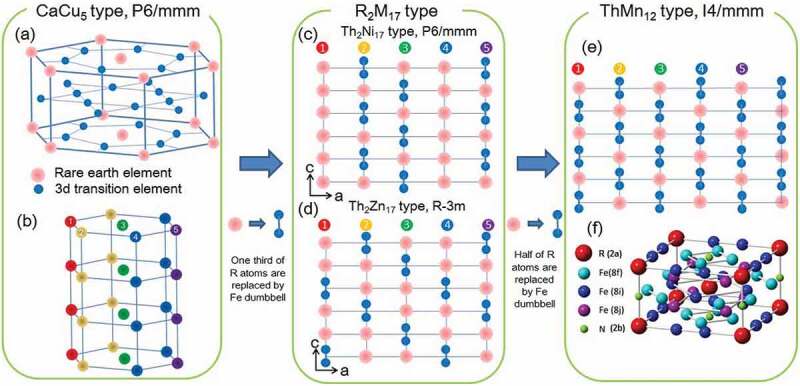
Schematic view of the crystal structures (a) CaCu_5_-type and (f) RFe_12_ with ThMn_12_-type. (b) Three CaCu_5_-type structures are stacked in the *c* direction. Atomic arrangement in the *c* direction of (c) Th_2_Ni_17_-type, (d) Th_2_Zn_17_-type, and (e) ThMn_12_-type

The RT_12_ phase has a ThMn_12_-type crystal structure with a space group of I4/mmm. Figure 1 shows schematic views of the crystal structures (a) CaCu_5_-type and (f) RFe_12_ with ThMn_12_-type. (b) Three CaCu_5_-type structures are stacked in the *c* direction. Atomic arrangement in the *c* direction are shown for (c) Th_2_Ni_17_-type, (d) Th_2_Zn_17_-type, and (e) ThMn_12_-type structures. These compounds are indexed by the compositional formula of R_n-m_T_5n+2m_, which *n* are 3, 2 or 1 and *m* is 1 or 0. The original crystal structure of ThMn_12_ is CaCu_5_-type, as shown in Figure 1(a), with a 1:5 ratio of rare-earth element (R) and 3d transition element (T) [32]. By substituting one-third of the R atoms with two T atoms, the R_2_T_17_ structure is obtained, as shown in Figure 1(c,d). These two T atoms are called ‘dumbbell’ because T atoms are aligned in the *c* direction and look like a dumbbell. There are two types of R_2_T_17_: Th_2_Ni_17_ and Th_2_Zn_17_. The R atoms are numbered from 1 to 5, as shown in Figure 1(b), where three of the CaCu_5_ structures are stacked in the *c* direction. The Th_2_Ni_17_-type structure shown in Figure 1(c) is formed by stacking the planes substituted by the 2^nd^ and 5^th^, and 3^rd^ R atoms with the T dumbbell. By stacking the planes substituted by the 2^nd^ and 5^th^, 3^rd^, and 1^st^ and 4^th^ R atoms with the T dumbbell, the Th_2_Zn_17_ type structure is obtained, as shown in Figure 1(d). The R_2_T_17_ compound with light and heavy rare-earth elements exhibited the Th_2_Zn_17_ and Th_2_Ni_17_ structures, respectively. When half of the R atoms are substituted with the T dumbbells, the ThMn_12_ structure is obtained, as shown in Figure 1(e). When R atoms are randomly substituted with T dumbbells, the TbCu_7_ structure is formed with a relatively wide composition with an R:T ratio ranging from 1:5 to 1:9 [33]. Figure 1(f) shows a schematic of the RFe_12_ compound with a ThMn_12_ crystal structure that is tetragonal. There are three different Fe sites – 8i, 8j, and 8f – and the site for the rare-earth element is 2a. The light elements can be interstitially located at the 2b sites.

One drawback of the RT_12_ phase with the ThMn_12_ structure is its instability at room temperature. According to Kobayashi et al., one of the possible reasons for its structural instability in the RFe_12_ phase is the excessively large interatomic distance between the two Fe atoms of a Fe–Fe dumbbell at the 8i site. The interatomic distances in the Fe sites are 0.271 nm, 0.259 nm, and 0.251 nm, respectively, for 8i, 8j, and 8f [34]. Compared with the atomic radius of Fe (~0.252 nm), the interatomic distance at the 8i site is larger. The larger interatomic distance at the 8i site causes an increase in the kinetic energy of electrons due to localization. Therefore, a larger distance in the Fe–Fe dumbbell destabilizes the structure. To stabilize the crystal structure, non-magnetic elements should be added. A trend can be observed by comparing the atomic radii of the phase stabilizing elements. As shown in Table 1, non-magnetic stabilizing elements with a larger atomic radius tend to substitute the 8i site, whose interatomic distance of the dumbbell is the largest in the structure. However, all the elements do not necessarily follow this trend. The theoretical calculations by Harashima et al. [35] show that, in elements with smaller atomic radii, the formation energies by the substitution of the phase stabilizing elements are similar for the three different Fe sites. This means that the phase stabilizing elements with a smaller atomic radius can substitute these three sites equally.

**Table 1.** Phase stabilizing elements, their atomic radii, and substitution sites

Phase stabilizing element	Atomic radius (nm)	Substitution site	References
Ti	0.147	8i	[1–5]
V	0.134	8i	[5–10]
Cr	0.128	8i,8j,8f	[5,11]
Mo	0.139	8i	[5,13,14]
Al	0.143	8j, 8f	[16]
Si	0.132	8j, 8f	[5,13,17]
Mn	0.127	8i,8j,8f	[19,20]
W	0.139		[18]
Zr	0.160	2a	[1]
Co	0.125	8j, 8f	[1–3]
Nb	0.146	8i	[21]
Ta	0.146	8i	[22]
Re	0.137		[18]
Fe	0.126	-	-
Sm	0.180	-	-

There is a stabilizing element that can substitute the R site to stabilize the ThMn_12_ structure. Kuno et al. [36] and Tozman et al. [37] reported that Zr stabilizes the ThMn_12_ structure and it substitutes the 2a site [9]. Moreover, Tozman et al. reported that the addition of Zr not only stabilized the ThMn_12_-type crystal structure but also enhanced the saturation magnetization of Sm(Fe_0.8_Co_0.2_)_12_ in the thin film form [38]. Because the atomic radius of R is very large, it imparts a significant amount of stress to the elements around it, which could be one of the reasons for the large distance between the Fe atoms at the 8i and 8j sites. By the substitution of the rare-earth element at the 2a site with an element having a smaller atomic diameter, the total energy would decrease. However, until now, only Zr is known as a stabilizing element for the 2a site to realize a ThMn_12_-type crystal structure.

Although there has been extensive research to realize a ThMn_12_-type crystal structure using various stabilizing elements, there is no universal understanding of the underlying physics on the phase stability owing to the substitution of the phase-stabilizing elements. Further investigation is necessary to fully understand the mechanism of phase stabilization by the addition of other elements. These fundamental investigations can provide guidelines on how to stabilize the ThMn_12_-type crystal structure with a minimum reduction of saturation magnetization while maintaining a larger magnetic anisotropy field and *T*_c_.

## 3. Synthesis and intrinsic properties of Sm(Fe-Co)_12_

**Figure 2.**
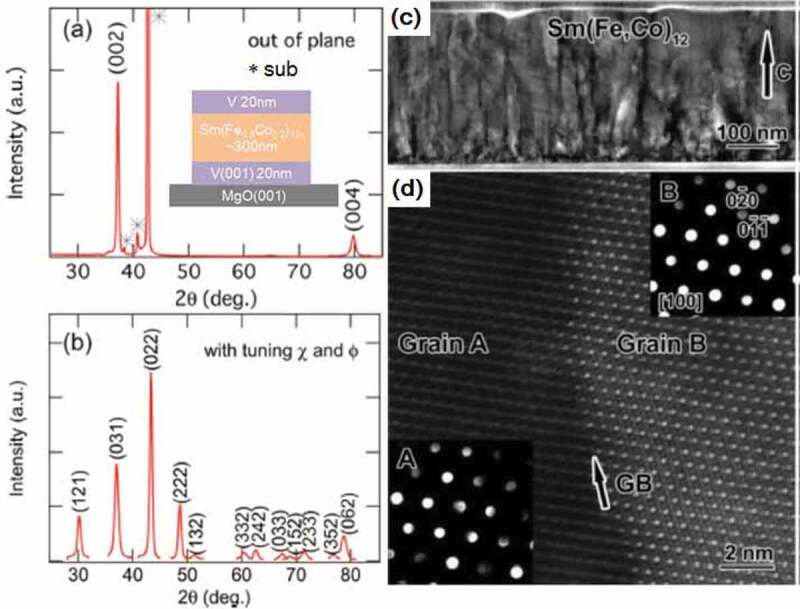
XRD patterns of (a) out-of-plane and (b) various planes with tuning the angle of the sample. Inset in (a) is the stacked film of the sample. (c) Cross-sectional bright-field TEM image, (d) magnified view of HAADF-STEM image and nano-beam electron diffraction NBED patterns measured at the two regions. The arrow in (c) represents the direction of the *c*-axis, and the arrow in (d) shows the grain boundary. Reproduced by permission from [31], copyright [2020, Elsevier]

In this section, we review the intrinsic properties of Sm(Fe-Co)_12_-based thin films to determine its potential as a permanent magnet [30,31]. Hirayama et al. successfully prepared Sm(Fe_1-x_Co_x_)_12_-based thin films with a ThMn_12_-type crystal structure by selecting an appropriate underlayer [30]. It was shown that Sm(Fe_0.8_Co_0.2_)_12_ shows a large saturation magnetization (µ_0_*M*_s_) of 1.78 T with an anisotropy field of 12 T and *T*_c_ of 859 K by depositing a ~ 600-nm thick film on the V underlayer. Ogawa et al. revisited this work and investigated in detail the intrinsic magnetic properties of the Sm(Fe_0.8_Co_0.2_)_12_ film [31]. As shown in Figure 2, the prepared sample shows the epitaxial growth of the ThMn_12_ structure. Although the cross-sectional bright-field transmission electron microscopy (TEM) image in Figure 2(c) shows a columnar structure, these grains have a ThMn_12_ structure with a slight misalignment, as shown in the nano-beam diffraction patterns in Figure 2(d).

**Figure 3.**
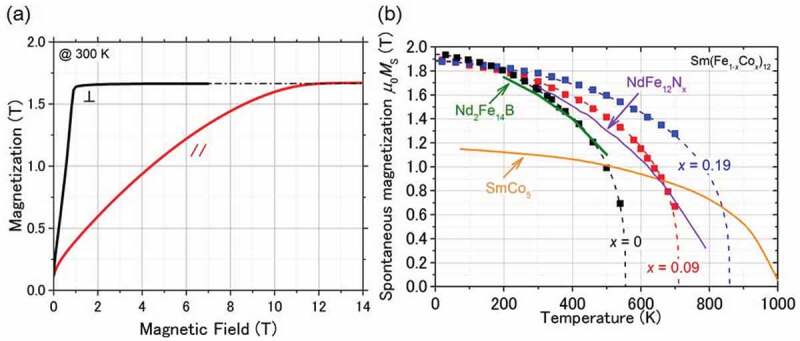
(a) Out-of-plane (black) and in-plane (red) magnetization curves of SmFe_12_ thin film. (b) Temperature dependence of the magnetization μ_0_*M*_s_ in Sm(Fe_1-x_Co_x_)_12_ thin films, where *x* = 0, 0.09, and 0.19. Reproduced by permission from [30], copyright [2020, Elsevier]

Figure 3 shows the magnetization curves of SmFe_12_ in the out-of-plane and in-plane directions of the film [31]. The film has a strong perpendicular anisotropy, which is consistent with the X-ray diffraction (XRD) results. The saturation field of the in-plane curve is approximately 12 T. However, *μ*_0_*H*_c_ is negligible owing to the no-pinning sites for magnetic domain wall motion in the continuous film. The *μ*_0_*M*_s_ is approximately 1.64 T, which is comparable to that of Nd_2_Fe_14_B. Figure 3(b) shows the temperature dependence of the magnetization of Sm(Fe_1-x_Co_x_)_12_ films [30]. The data for Nd_2_Fe_14_B, SmCo_5_, and NdFe_12_N_x_ are also plotted in the same figure. With increasing Co content (*x*), *T*_c_ increases from 555 K for *x* = 0 to 859 K for *x* = 0.2, which is higher than that of Nd_2_Fe_14_B. Although the *T*_c_ of SmCo_5_ is much larger than that of the Sm(Fe_0.8_Co_0.2_)_12_ film, the *μ*_0_*M*_s_ shows a higher value for Sm(Fe_0.8_Co_0.2_)_12_. *μ*_0_*M*_s_ increases with *x*, 1.64 T for *x* = 0 to 1.78 T for *x* = 0.19 at room temperature [30].

**Figure 4.**
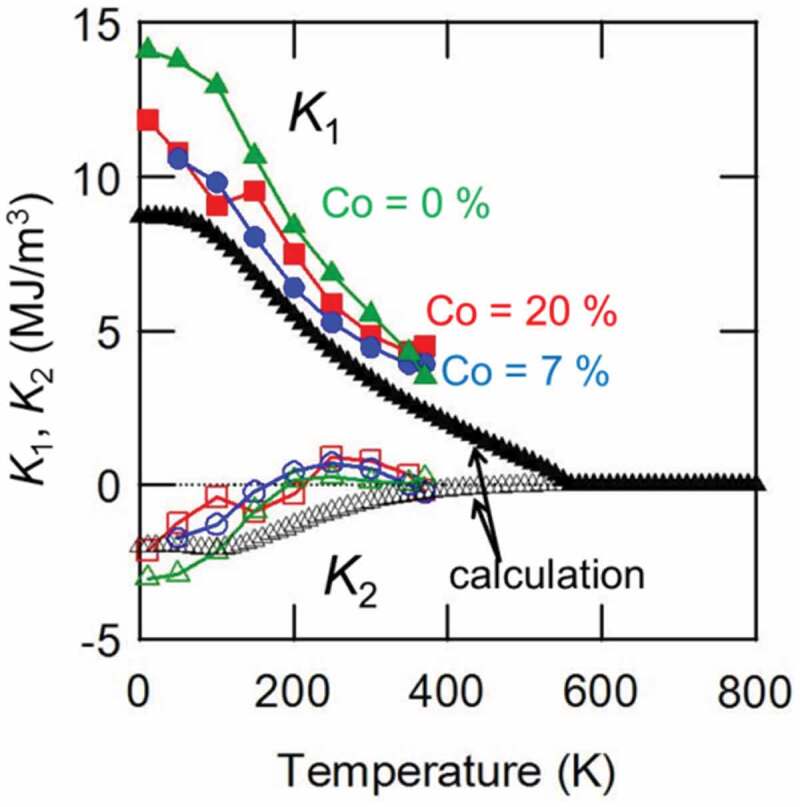
First and second-order uniaxial magnetic anisotropy constants *K*_1_ and *K*_2_ for Sm(Fe_1-x_Co_x_)_12_ films (*x* = 0, 0.07, 0.2) as a function of temperature. Reproduced by permission from [31], copyright [2020, Elsevier]

Ogawa et al. measured the temperature dependence of the first- and second-order uniaxial magnetic anisotropy constants, *K*_1_ and *K*_2_, of the Sm(Fe_1-x_Co_x_)_12_ film, as shown in Figure 4 [31]. *K*_1_ and *K*_2_ were estimated using the AHE-torque method [39]. The theoretically calculated results are shown in the same figure. In all the films having different *x* values, *K*_1_ decreases monotonically with temperature. However, *K*_2_ is negative at temperatures lower than 200 K and shows a small maximum around 300 K. Negative *K*_2_ value makes the upward deviation from a linear extrapolation of the low field part in the hard-axis magnetization curve. The absolute value of *K*_1_ is the largest for the SmFe_12_ film, which is decreased by the substitution of Co for Fe sites.

The intrinsic properties of Sm(Fe_1-x_Co_x_)_12_ are summarized in Table 2. Sm(Fe_0.8_Co_0.2_)_12_ shows high intrinsic magnetic properties of *μ*_0_*M*_s_ = 1.78 T, *μ*_0_*H*_a_ = 8.20 T, and *T*_c_ = 859 K at room temperature [30], which are superior to those of Nd_2_Fe_14_B (*μ*_0_*M*_s_ = 1.61 T, *μ*_0_*H*_a_ = 7.5 T, and *T*_c_ = 585 K at room temperature) [1] and the commercial Nd_2_Fe_14_B magnet of N50 (*μ*_0_*B*_r_ = 1.44 T and *T*_c_ = 593 K at room temperature) [40].

**Table 2.** Intrinsic properties of Sm(Fe_1-x_Co_x_)_12_. Numbers in parentheses are anisotropy field μ_0_*H*_a_ estimated by magnetization curves. Reproduced by permission from [30], copyright [2020, Elsevier]

	@300 K					@0 K		
x	μ_0_*M*_s_ (T)	μ_0_*H*_a_ (T)	*K*_1_ (MJ/m^3^)	*K*_2_ (MJ/m^3^)	*T*_c_ (K)	μ_0_*M*_s_ (T)	s	D (meV/Å^2^)
0.00	1.64	7.62 (12)	5.56	0.11	555	1.94	1.01	179
0.09	1.71	7.54 (12)	4.45	0.53	710	1.89	0.90	251
0.19	1.78	8.20 (12)	4.37	0.71	859	1.88	0.73	351

## 4. *μ*_0_*H*_c_ enhancement by the diffusion process and a light element inclusion

Investigations on the intrinsic magnetic properties of SmFe_12_-based compounds in the thin film form showed that Sm(Fe_0.8_Co_0.2_)_12_ has the potential for the next generation of permanent magnets because of its high *μ*_0_*M*_s_, *K*, and *T*_c_. However, unless these intrinsic magnetic properties are not transferred into the extrinsic ones, in particular, a large remanent magnetization and sufficiently large coercivity (µ_0_*H*_c_ > µ_0_*M*_s_/2 > 0.9 T), SmFe_12_-based compounds cannot be realized as practical permanent magnets. Since *μ*_0_*H*_c_ is an extrinsic magnetic property and is governed by realizing an optimum microstructure, microstructural control is the key for a high *μ*_0_*H*_c_. *μ*_0_*H*_c_ shows the difficulty in the nucleation and/or movement of magnetic domain walls in magnetic materials for practical applications. Therefore, control over different microstructural factors such as the grain size and grain boundary phase is necessary. The latter can act as pinning sites against magnetic domain wall motion. In addition, because high *μ*_0_*M*_s_ is also required for a stronger magnet, a smaller volume fraction of the non-magnetic phase is preferable. Considering these two requirements, the ideal microstructure for the permanent magnet is a nanogranular structure with small and hard magnetic grains enveloped by a thin non-magnetic phase. One example of a magnet possessing the ideal microstructure is the Nd–Fe–B magnet. Several hundred nanometers of Nd_2_Fe_14_B grains are exchange-decoupled by Nd-rich phases, which result in a *μ*_0_*H*_c_ of above 2 T [41–43]. The advantage of Nd–Fe–B magnets is that Nd_2_Fe_14_B and Nd-rich phases are in thermodynamic equilibrium [44]. However, as mentioned in the introduction, the RT_12-x_Ti_x_ system does not have such non-magnetic phases that can be equilibrated with the RT_12_ phase. In Nd–Fe–B magnets, the infiltration of Nd–Cu eutectic alloy to the grain boundary of Nd_2_Fe_14_B significantly enhanced the *μ*_0_*H*_c_ [41–43]. However, such a microstructure has not been attained in SmFe_12_-based systems. Since control of the microstructure is rather easy in the thin film form, several efforts have been made to realize this optimum microstructure in SmFe_12_-based thin films. Ogawa et al. used the diffusion process to enhance *μ*_0_*H*_c_ in Sm(Fe_0.8_Co_0.2_)_12_ films [45].

**Figure 5.**
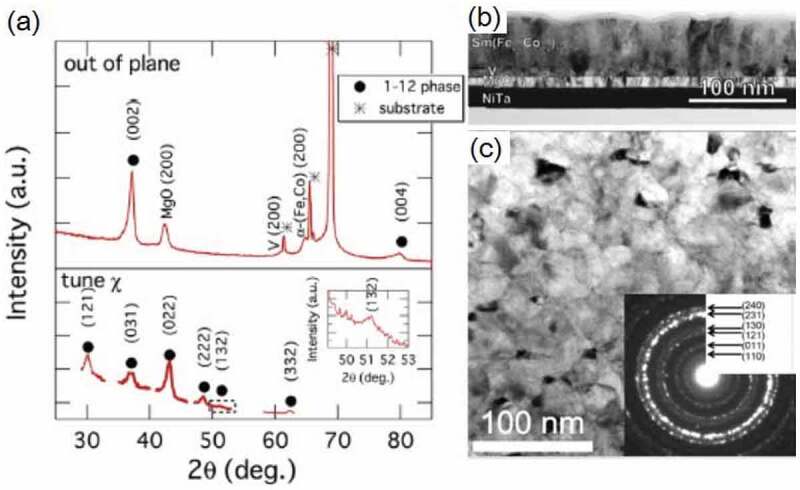
(a) XRD patterns of the out-of-plane and various planes for the as-deposited Sm(Fe_0.8_Co_0.2_)_12_ thin film. (b) Cross-sectional bright-field TEM image, and (c) in-plane bright-field TEM image of the as-deposited Sm(Fe_0.8_Co_0.2_)_12_ thin film. Reproduced by permission from [45], copyright [2020, Elsevier]

**Figure 6.**
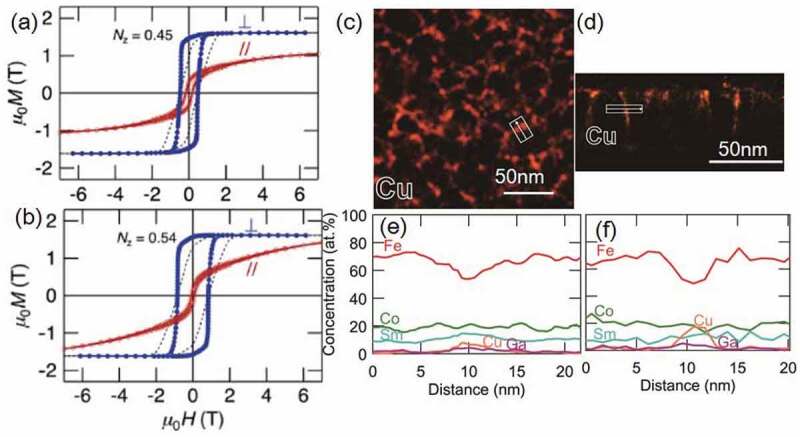
Out-of-plane and in-plane magnetization curves with demagnetization field correction for (a) the as-deposited anisotropic polycrystalline Sm(Fe_0.8_Co_0.2_)_12_ thin film and (b) Cu–Ga diffused Sm(Fe_0.8_Co_0.2_)_12_ thin film. Elemental maps of Cu in the (c) plane view and (d) cross-sectional view. Energy dispersive X-ray spectroscopy (EDS) line scan profiles obtained from the white rectangular region in the Cu map across the grain boundary (e) in-plane view, and (f) in cross-sectional view for the Cu–Ga deposited and post-annealed Sm(Fe_0. 8_Co_0.2_)_12_ thin film. Reproduced by permission from [45], copyright [2020, Elsevier]

The infiltration of the non-magnetic alloys into the large angle grain boundaries with less lattice coherency in the adjacent grains can be easier than that of small angle grain boundaries. Hence, Ogawa et al. used a thermally oxidized Si substrate having an amorphous surface and prepared an Sm(Fe_0.8_Co_0.2_)_12_ layer on top of the NiTa/MgO/V underlayers [30]. Here, NiTa is amorphous and MgO grows naturally on the lowest energy (001) plane. Subsequently, the V layer was epitaxially grown on the (001) textured MgO layer. As shown in Figure 5(a), the Sm(Fe_0.8_Co_0.2_)_12_ film is highly (001) textured, and the superlattice peaks of (132) and (332) are observed. Figure 5(b,c) show the cross-sectional and in-plane bright-field TEM images of the highly (001) textured polycrystalline sample. The average grain size is approximately 50 nm, and a columnar structure is observed. Ogawa et al. carried out an infiltration process using different infiltration materials to form grain boundaries isolating a highly (001) textured polycrystalline Sm(Fe_0.8_Co_0.2_)_12_ grains in the thin film form [45]. As shown in a figure later (Figure 10), several materials show enhanced *μ*_0_*H*_c_ due to the infiltration process. A detailed analysis of the Cu–Ga diffused sample is given in Figure 6. Cu–Ga infiltrated film shows a high *μ*_0_*H*_c_ of 0.84 T. Figure 6(a,b) show the magnetization curves before and after the infiltration process of Cu–Ga. Before the infiltration process, the film has a strong perpendicular anisotropy, which results in the highly (001) texture, and *μ*_0_*H*_c_ is approximately 0.48 T. After the infiltration process using Cu–Ga, *μ*_0_*H*_c_ increases to 0.84 T. Figure 6(c) shows the in-plane elemental mapping of the Cu–Ga infiltrated sample, where Cu is found to be segregated in the grain boundaries. Figure 6(e) shows the composition profile obtained from the rectangular region in the Cu map across the grain boundary. Fe is depleted and Cu and Ga are enriched in the grain boundary. The compositions of the main phase and grain boundary are Sm(Fe_0.77_Co_0.2_Cu_0.01_Ga_0.02_)_10.6_, and Sm(Fe,Co,Cu,Ga)_z_(z = 6.19.0), respectively. Figure 6(d) shows the cross-sectional elemental maps of Cu. Cu diffuses into the grain boundaries. The concentration profiles calculated from the elemental map are shown in Figure 6(f). This grain boundary phase is considered to be either a TbCu_7_- or TbCu_9_-type structure based on the composition [46] that exhibit different magnetization and anisotropy constants from those of the main Sm(Fe_0.8_Co_0.2_)_12_ phase. This phase affects *μ*_0_*H*_c_ because it can act as the pinning site of domain walls or weaken inter-grain exchange coupling. Low eutectic temperature alloys of Al–Zn, Mg–Zn, and Sn–Zn are effective in increasing *μ*_0_*H*_c_ at low temperature of infiltration process, as shown in Figure 10. Ogawa et al. reported the highest *μ*_0_*H*_c_ of 0.87 T using Mg–Zn as the infiltration source after annealing at 673 K.

The temperature coefficient of *μ*_0_*H*_c_, *β*, describes the thermal stability of *μ*_0_*H*_c_, which is defined as

$$\beta =\frac{\mu_{0}H_{c}\left(T\right)-\mu_{0}H_{c}\left(300\right)}{\mu_{0}H_{c}\left(300\right)\cdot \left(T-300\right)}$$

where *μ*_0_*H*_c_(300) and *μ*_0_*H*_c_(T) denote *μ*_0_*H*_c_ at 300 K and temperature *T*, respectively. Ogawa et al. reported a *β* of −0.20%/K (300–650 K) in a Cu–Ga-infiltrated sample [45]. The *β* values are better than those of commercial Nd–Fe–B sintered magnets (*β* = −0.6 to −0.43%/K) [47,48], while they are comparable to those of Sm(Fe,Co)_z_-based magnets with RT_7_ or R_2_T_17_ structure (*β* = −0.2 to −0.16%/K) [49].

**Figure 7.**
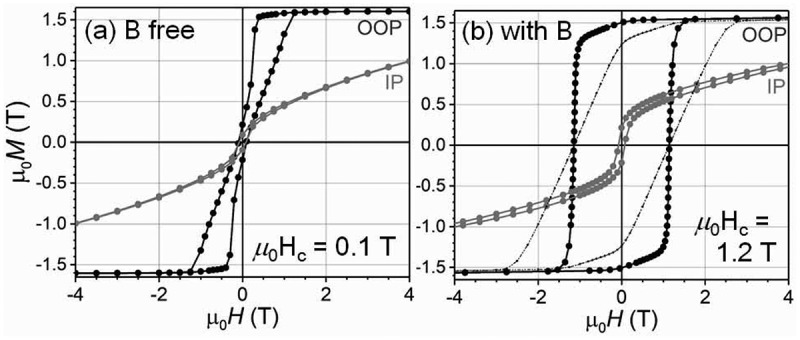
Out-of-plane and in-plane magnetization curves of (a) B-free and (b) B-containing samples [50]

**Figure 8.**
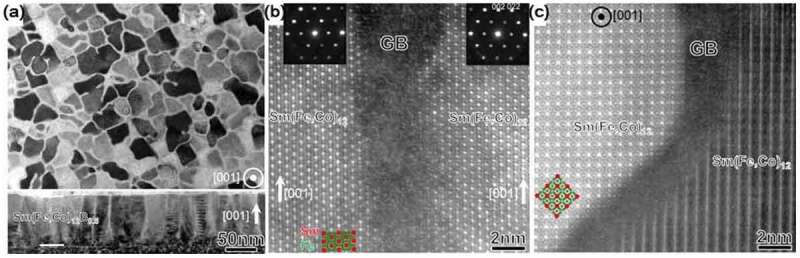
Microstructures of Sm(Fe_0.8_Co_0.2_)_12_B_0.5_. (a) In-plane and cross-sectional bright-field TEM images, and high-resolution HAADF-STEM images in the (b) cross-sectional and (c) in-plane directions [50]

**Figure 9.**
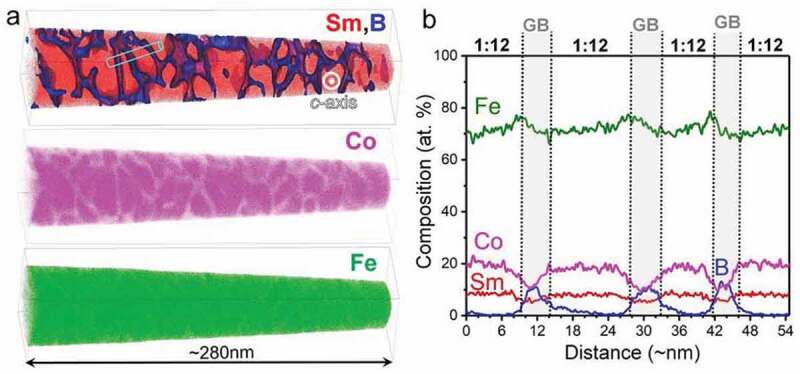
(a) Atom maps of Sm&B, Co, and Fe as well as the isosurface of the B-rich region in the Sm(Fe_0.8_Co_0.2_)_12_B_0.5_ thin film. (b) Composition diagram in the box in the Sm&B map is shown in (a) [50]

Another approach to achieve a high *μ*_0_*H*_c_ is by the co-sputtering of a non-magnetic element that does not form a solid solution with Sm(Fe_0.8_Co_0.2_)_12_. Sepehri-Amin et al. showed that co-sputtering of B with Sm(Fe_0.8_Co_0.2_)_12_ thin films resulted in a substantial increase in coercivity of ~1.2 T [50]. Figure 7 shows the magnetization curves for Sm(Fe_0.8_Co_0.2_)_12_ and Sm(Fe_0.8_Co_0.2_)_12_B_0.5_ films. Both films show strong perpendicular anisotropy. The B-free sample shows a small *μ*_0_*H*_c_ of approximately 0.1 T, while the coercivity of B-containing sample is 1.2 T. In addition to the high *μ*_0_*H*_c_, a high remanent magnetization (*μ*_0_*M*_r_) of 1.5 T is realized in the B-containing Sm(Fe_0.8_Co)_12_B_0.5_ film [50]. To understand the mechanism of large *μ*_0_*H*_c_ by the addition of B, Sepehri-Amin et al. observed the microstructure of the samples. Figure 8(a) shows the in-plane and cross-sectional bright-field TEM images of the Sm(Fe_0.8_Co_0.2_)_12_B_0.5_ film. In the B-free Sm(Fe_0.8_Co_0.2_)_12_ sample, similar to what was shown in Figure 2(c), the columnar grains can be grown while they are in direct contact, as discussed in detail by Sepehri-Amin et al. [50]. By the addition of B, a nanogranular structured Sm(Fe_0.8_Co_0.2_)_12_ grains with an average diameter of ~50 nm that is enveloped by a grain boundary phase can be observed [50]. Figure 8(b,c) show the high-resolution high-angle annular dark-field scanning transmission electron microscopy (HAADF-STEM) images in the cross-section and in-plane directions. An amorphous grain boundary phase with a thickness of approximately 3 nm separated the Sm(Fe_0.8_Co_0.2_)_12_ grains in the film having 1.2 T coercivity [50]. To understand the chemistry of the grain boundary and mechanism of the *μ*_0_*H*_c_ enhancement, Sepehri-Amin et al. carried out a more detailed microstructural analysis using a three-dimensional atom probe (3DAP) [50]. Figure 9(a) shows the atom maps of Sm&B, Co, and Fe as well as the isosurface of the B-rich region in the Sm(Fe_0.8_Co_0.2_)_12_B_0.5_ film. The analysis direction was parallel to the film plane. A composition line profile along the box in the Sm&B map is shown in Figure 9(b). Although the composition of Fe does not change appreciably in the grain boundary, B is enriched and Co is depleted. Sm slightly decreased in the grain boundary. The composition of B in the grain boundary is approximately 10.3%. The composition of the 1–12 phase and the amorphous intergranular phase are Sm_8.0_Fe_71.9_Co_19.8_B_0.3_ and Sm_5.8_Fe_73.3_Co_10.6_B_10.3_, respectively. Since the composition of Fe and Co in the grain boundary phase is approximately 84%, it is thought to be a ferromagnetic phase. The large difference in the magnetic anisotropy between the magnetically hard Sm(Fe_0.8_Co_0.2_)_12_ grains and the soft Fe-Co-B amorphous phase acts as the pinning site for magnetic domain wall motion. A thin intergranular phase with a ferromagnetic nature covering 1:12 grains can act as pinning sites against magnetic domain wall propagation during the magnetization reversal process. The influence of thin intergranular phase against magnetic domain wall propagation has been addressed by micromagnetic simulations based on the microstructures of Nd-Fe-B and Sm_12_Fe_74_V_12_Cu_2_ systems [51–54]. The pinning effect is active only when the width of the ferromagnetic grain boundary is less than two times of exchange length 2 × *l*_ex_ of Nd_2_Fe_14_B matrix phase. The latter is necessary to hinder the nucleation of reversed magnetic domains from the ferromagnetic grain boundary phase [51–54]. In addition to the high *μ*_0_*H*_c_, the Sm(Fe_0.8_Co_0.2_)_12_B_0.5_ film shows excellent thermal stability of coercivity (*β* = −0.22%/K), which is superior to that of other high-performance commercial permanent magnets [31]. Note that Alnico magnets have a higher temperature coefficient of coercivity, but its hard magnetic properties are not attractive for high-performance permanent magnet applications. The large remanent magnetization, sufficiently large coercivity, and excellent thermal stability of coercivity in the Sm(Fe_0.8_Co_0.2_)_12_B_0.5_ film demonstrate the potential of Sm(Fe_0.8_Co)_12_ compounds for the development of high-performance permanent magnets if this optimum microstructure can be developed also in the bulk form.

**Figure 10.**
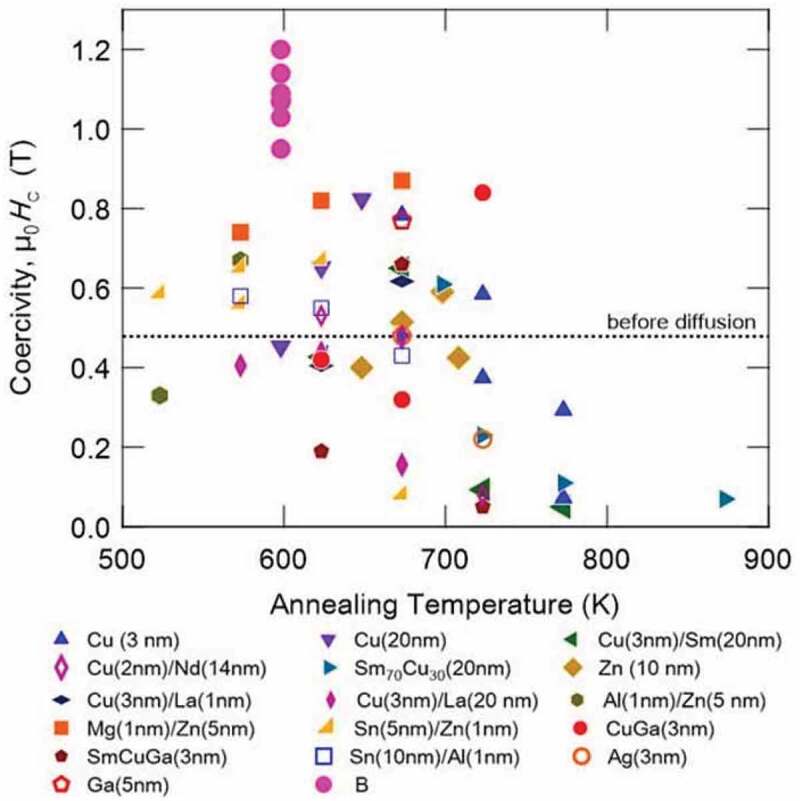
Coercivity (μ_0_*H*_c_) in the Sm(Fe_0.8_Co_0.2_)_12_-based thin films after the diffusion process. B was introduced by co-sputtering. Reproduced by permission from [43], copyright [2020, Elsevier]

**Figure 11.**
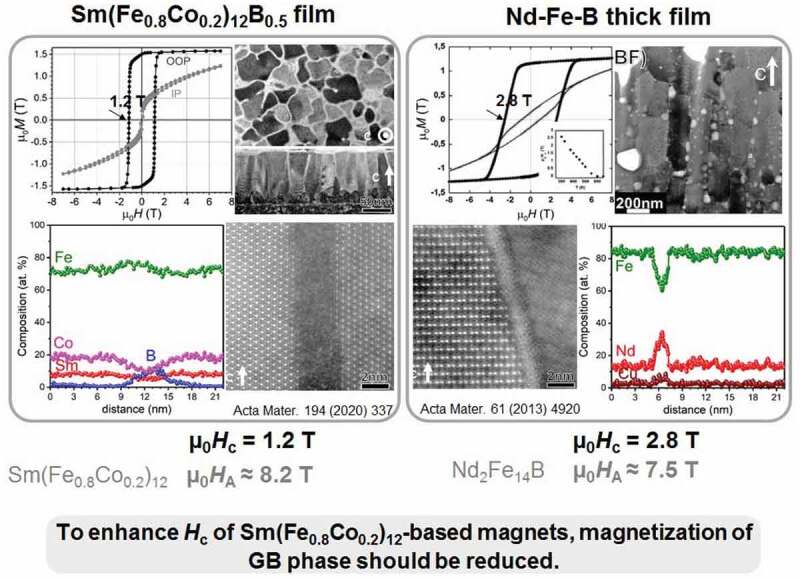
Comparison of the magnetic properties and microstructures in Sm(Fe_0.8_Co_0.2_)_12_B_0.5_ and Nd–Fe–B films. Reproduced by permission from [50,55], copyright [2020, Elsevier]

Figure 10 shows a summary of *μ*_0_*H*_c_ in the Sm(Fe_0.8_Co_0.2_)_12_-based thin films. Relatively high *μ*_0_*H*_c_ ~ 0.8 T was achieved by the Ga–Cu infiltration process. Ogawa et al. reported that further enhancement of *μ*_0_*H*_c_ could be achieved by further microstructural modifications in the infiltration processed films because the grain boundary phase did not reach the base of the film [45]. The most effective element in the enhancement of *μ*_0_*H*_c_ is B. The maximum *μ*_0_*H*_c_ attained by the addition of B is 1.2 T. However, considering the magnetic anisotropy field of Sm(Fe_0.8_Co_0.2_)_12_ (µ_0_*H*_A_ ≈ 8.2 T), the obtained coercivity in the Sm(Fe_0.8_Co_0.2_)_12_B_0.5_ film is only 15% of the anisotropy field. Figure 11 compares the magnetic properties and overall microstructure of the Sm(Fe_0.8_Co_0.2_)_12_B_0.5_ film with that of the Nd–Fe–B thick film reported by Dempsey et al. [55]. Although the grains in both films are columnar shaped with a similar aspect ratio, the grain size in the Sm(Fe_0.8_Co_0.2_)_12_B_0.5_ film is 4 times smaller. High-resolution STEM-HAADF images show that the grain boundaries in the Sm(Fe_0.8_Co_0.2_)_12_B_0.5_ film and Nd–Fe–B thick film are amorphous. However, the grain boundary phase in the Sm(Fe_0.8_Co_0.2_)_12_B_0.5_ film is slightly thicker (~3 nm) than that of the Nd–Fe–B thick film (~2 nm). The main difference is the composition of the grain boundaries, as shown in Figure 11. The grain boundary phase in the Sm(Fe_0.8_Co_0.2_)_12_B_0.5_ film has a large amount of Fe (~72%) while the Fe content in the grain boundary phase of the Nd–Fe–B thick film is reduced to ~60% suggesting the grain boundary phase in the Nd–Fe–B thick film is a weak ferromagnetic phase. The coercivity of the Nd–Fe–B thick film is reported to be 2.8 T, which is approximately 37% of the anisotropy field of the Nd_2_Fe_14_B phase (*μ*_0_*H*_A_ ≈ 7.5 T) [55]. This comparison shows that a much larger coercivity is expected in Sm(Fe_0.8_Co_0.2_)_12_-based magnets if a weak or non-ferromagnetic grain boundary phase is realized by isolating the Sm(Fe_0.8_Co_0.2_)_12_ grains.

## 5. Recent progress in bulk magnets in RT_12_-based systems

Sm(Fe_0.8_Co_0.2_)_12_-based thin films with a ThMn_12_-type crystal structure can be developed without any stabilizing element if an appropriate underlayer is used. However, stabilizing elements are needed in the bulk form to realize a ThMn_12_-type crystal structure. Therefore, the question is whether the intrinsic magnetic properties of Sm(Fe,Co)_12-x_M_x_ compounds (M = stabilizing element) can outperform that of the Nd_2_Fe_14_B phase.

Kuno et al. investigated the intrinsic magnetic properties of the strip cast (Sm,Zr)(Fe,Co)_11.5_Ti_0.5_ powders with a high *μ*_0_*M*_s_ of 1.63 T, magnetic anisotropy field (*μ*_0_*H*_a_) of 7.4 T at room temperature, and *T*_c_ of approximately 880 K [36]. High *μ*_0_*M*_s_ has been achieved by reducing the amount of the phase stabilizing element Ti. The addition of Zr and Co improved the phase stability of this Ti-lean sample. Tozman et al. demonstrated that intrinsic magnetic properties (*μ*_0_*H*_a_ and *μ*_0_*M*_s_) of Sm(Fe_0.8_Co_0.2_)_11_Ti and Sm_0.8_Zr_0.2_(Fe_0.8_Co_0.2_)_11.5_Ti_0.5_ alloys are superior to those of Nd_2_Fe_14_B phase at elevated temperatures [37]. Another issue for realizing Sm(Fe_0.8_Co_0.2_)_12_-based bulk magnet is the establishment of an optimum microstructure to realize a large *μ*_0_*M*_r_ and sufficient *μ*_0_*H*_c_.

**Figure 12.**
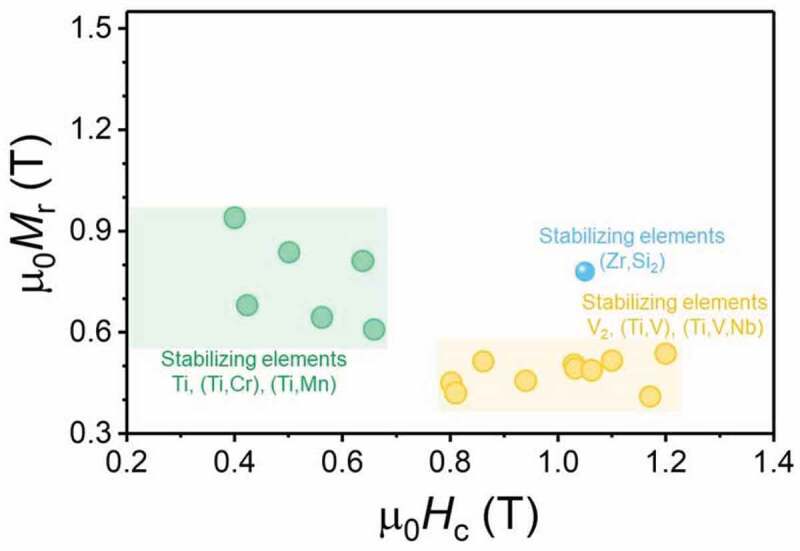
Remanent magnetization versus coercivity µ_0_*H*_c_ of various SmFe_12_-based compounds with various stabilizing elements [56–60]

**Figure 13.**
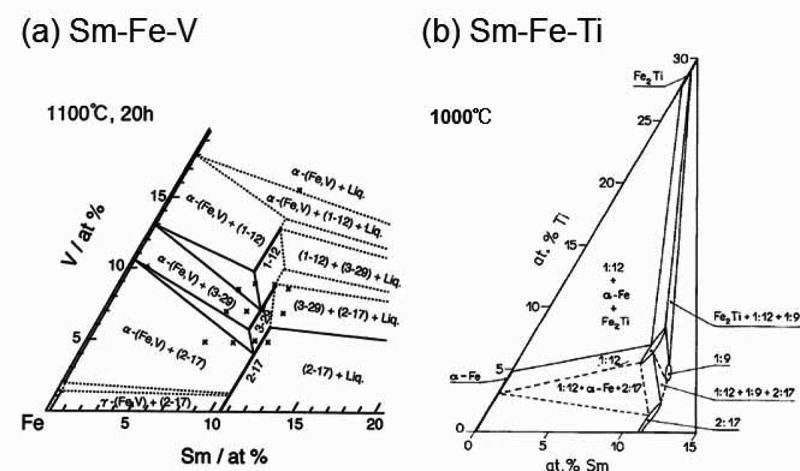
Phase diagrams of (a) Sm-Fe-V [62] and (b) Sm-Fe-Ti [28]

There have been several efforts to realize coercivity in SmFe_12_-based compounds. The main approach has been the reduction of the grain size by rapid solidification, mechanical alloying, and mechano-chemical processes. Figure 12 shows the remanent magnetization versus *μ*_0_*H*_c_ of various SmFe_12_-based compounds with different stabilizing elements [56–60]. Except for (Sm_0.7_Zr_0.3_)Fe_10_Si_2_ alloys, which are prepared by a mechano-chemical process, all the alloys with different stabilizing elements prepared by mechanical alloying or melt-spinning are isotropic. A sufficiently large coercivity of ~1.2 T can be realized only when V is used as one of the stabilizing elements. The required V content to stabilize ThMn_12_-type crystal structure in the RFe_12-X_M_X_ system is almost two times larger than the required amount of Ti, which results in a larger reduction of *μ*_0_*M*_s_ [61]. However, the coercivity of Ti-containing and V-free alloys are below ~0.65 T. Recent microstructural investigations by Schӧnhӧbel et al. on the hot-deformed Sm_12_Fe_74_V_12_Cu_2_ magnets prepared from mechanically alloyed powders showed that the formation of Sm-rich and Fe-lean grain boundary phase is responsible for realizing coercivity [51]. In the ternary-phase diagram of Sm–Fe–V at 1100°C shown in Figure 13(a), the 1–12 phase is in equilibrium with a 3–29 and an Sm-rich liquid phase [62]. Hence, an Sm-rich grain boundary phase was observed in the 1–12 magnet with V as the stabilizing element. In the case of the Sm–Fe–Ti ternary-phase diagram shown in Figure 13(b), a liquid phase or non-ferromagnetic phase, which is not in equilibrium with the 1–12 phase can be used as the grain boundary phase with isolated 1–12 grains. This can be one of the reasons that no large coercivity has been realized in SmFe_11_Ti-based alloys. Moreover, the magnetization of the reported 1–12 powders with a coercivity of ~1.0–1.2 T is low because of the large amount of stabilizing elements. The alloy composition of the reported SmFe_12_-based powders or hot-pressed magnets that show some coercivity do not contain Co. Based on the report by Tozman e*t al., μ*_0_*M*_s_ can be enhanced in Sm(Fe_1-x_Co_x_)_11_Ti alloys without the anisotropy field in the case of *x* ≤ 0.2 [37]. Hence, the addition of Co can be another approach to enhance *μ*_0_*M*_s_.

For the development of anisotropic bulk 1–12 magnets, Dirba et al. developed jet-milled Sm(Fe_0.8_Co_0.2_)_11_Ti, Sm(Fe_0.8_Co_0.2_)_10.5_TiGa_0.5_, and Sm(Fe_0.8_Co_0.2_)_10.5_TiCu_0.5_ powders with a fine average particle size of ~5 µm from the alloys produced by induction melting. Dirba et al. reported that the trace addition of Ga and Cu is beneficial for the realization of the Sm–Ga or Sm–Cu rich intergranular phase with excellent wettability at the 1–12 grain surface [63]. Trace addition element should fulfill the conditions of good wettability, small solubility to 1–12 grains and low-melting points in order to form the grain boundary phase. However, annealing of the jet-milled powders at temperatures above 600°C resulted in surface decomposition, which occurred by the evaporation of Sm and the remaining Fe and Fe_2_Ti phases at the powder surface, which became more pronounced upon reduction of the particle size [64] and are detrimental for coercivity. The evaporation of Sm is a more serious problem in sintering process over 1000°C. This could limit the use of ultra-fine particles as a precursor for the development of bulk anisotropic magnets via the liquid sintering process. In order to obtain a sufficiently large coercivity, surface oxidation should be suppressed. The hydrogenation–disproportionation–desorption–recombination (HDDR) process widely used for Nd–Fe–B-based magnets is another known method to reduce the grain size to ~250 nm, while the particle size remains as large as 50–300 µm. Based on the reports on the HDDR-processed Nd–Fe–B powders, the optimum HDDR processing condition can result in the development of anisotropic powders [65–67]. Dirba et al. employed the HDDR process and developed ultra-fine grain-sized Sm(Fe_0.8_Co_0.2_)_11_Ti-based powders [63]. Although ThMn_12_-type crystal structure was realized under an optimum processing condition, the powders did not show any large coercivity even by trace addition of other elements such as Ga and Cu [68]. Moreover, the developed HDDR process was isotropic, and only local texture between 1–12 grains and Fe_2_Ti was observed during the HDDR process. The main reason for the low coercivity in the HDDR-processed SmFe_12_-based powders was the lack of a weak or non-ferromagnetic grain boundary phase to magnetically isolate 1–12 grains. Hot-press and hot-deformation of rapidly solidified powders is a well-known method to develop anisotropic bulk hot-deformed Nd-Fe-B magnets [69,70]. However, developed hot-deformed bulk SmFe_12_-based magnets show a limited degree of texture as discussed by Schönhöbel et al [51,71]. Hence, conventional hot-press and hot-deformation process is not an ideal method to develop anisotropic bulk SmFe_12_-based magnets.

Based on these investigations, realizing an optimum grain boundary phase that can magnetically isolate 1–12 grains or act as pinning sites against magnetic domain wall motion is the main bottleneck toward the development of bulk SmFe_12_-based magnets with higher performance. A recent report by Otsuka et al. showed the successful realization of a bulk anisotropic SmFe_10_V_2_ magnet with a coercivity of 0.8 T by conventional powder processing and liquid sintering [72]. This coercivity is low and is only 8% of the magnetic anisotropy field of the SmFe_10_V_2_ phase [73]. Moreover, the remanent magnetization was limited to 0.73 T because a large amount of V was necessary to stabilize the ThMn_12_ phase. Minimizing the stabilizing elements, revisiting the phase diagram, and expanding our understanding of the quaternary Sm–Fe–Ti–X systems are necessary to realize an equilibrated grain boundary phase. Moreover, a good wettability with the ThMn_12_ phase, which can be used as a grain boundary phase to envelope 1–12 grains, is needed to develop anisotropic bulk magnets with a sufficiently large coercivity.

## 6. Summary

We have reviewed recent progress in the development of SmFe_12_-based permanent magnets. Recent investigations on Sm(Fe_0.8_Co_0.2_)_12_ compounds in thin film form have shown its potential for permanent magnet applications owing to its high intrinsic magnetic properties – *μ*_0_*M*_s_, *K*, and *T*_c_. Even at 150°C, which is the working temperature of the magnet for the motor in EVs/HEVs, the intrinsic magnetic properties are superior to those of the Nd–Fe–B magnet. In addition to these high intrinsic properties, a high *μ*_0_*H*_c_ of 1.2 T was demonstrated by the addition of B in the thin film. The microstructural origin for this large coercivity was ascribed to the development of a nanogranular microstructure that consists of Sm(Fe_0.8_Co_0.2_)_12_ grains of size ~50 nm that is enveloped by B-rich amorphous grain boundaries. However, the obtained coercivity in the thin film form, as a model microstructure, is only 15% of the anisotropy field of Sm(Fe_0.8_Co_0.2_)_12_ compound, and a much larger coercivity is expected if the magnetism of the grain boundary phase can be modified to that of a weak ferromagnetic phase. This large coercivity was only realized in isotropic bulk magnets with small magnetization due to the isotropic nature of the magnet as well as the use of large amount of stabilizing elements. To realize this large coercivity while maintaining a large magnetization in an anisotropic SmFe_12_-based bulk permanent magnet, the first step is to minimize the stabilizing elements. Moreover, revisiting the phase diagram and expanding our understanding of a novel system in which a liquid phase can be in equilibrium with SmFe_12_-based grains to form weak- or non-ferromagnetic grain boundary phases are necessary to attain sufficiently large coercivity in anisotropic bulk SmFe_12_-based magnets.
